# Early prenatal alcohol exposure alters imprinted gene expression in placenta and embryo in a mouse model

**DOI:** 10.1371/journal.pone.0197461

**Published:** 2018-05-15

**Authors:** Heidi Marjonen, Mia Toivonen, Laura Lahti, Nina Kaminen-Ahola

**Affiliations:** 1 Department of Medical and Clinical Genetics, Medicum, University of Helsinki, Helsinki, Finland; 2 Department of Biological and Environmental Sciences, Division of Genetics, University of Helsinki, Helsinki, Finland; Institute of Molecular Genetics of Montpellier, FRANCE

## Abstract

Prenatal alcohol exposure (PAE) can harm the embryonic development and cause life-long consequences in offspring’s health. To clarify the molecular mechanisms of PAE we have used a mouse model of early alcohol exposure, which is based on maternal *ad libitum* ingestion of 10% (v/v) ethanol for the first eight days of gestation (GD 0.5–8.5). Owing to the detected postnatal growth-restricted phenotype in the offspring of this mouse model and both prenatal and postnatal growth restriction in alcohol-exposed humans, we focused on imprinted genes *Insulin-like growth factor 2* (*Igf2)*, *H19*, *Small Nuclear Ribonucleoprotein Polypeptide N* (*Snrpn*) and *Paternally expressed gene 3* (*Peg3)*, which all are known to be involved in embryonic and placental growth and development. We studied the effects of alcohol on DNA methylation level at the *Igf2/H19* imprinting control region (ICR), *Igf2* differentially methylated region 1, *Snrpn* ICR and *Peg3* ICR in 9.5 embryonic days old (E9.5) embryos and placentas by using MassARRAY EpiTYPER. To determine alcohol-induced alterations globally, we also examined methylation in long interspersed nuclear elements (Line-1) in E9.5 placentas. We did not observe any significant alcohol-induced changes in DNA methylation levels. We explored effects of PAE on gene expression of E9.5 embryos as well as E9.5 and E16.5 placentas by using quantitative PCR. The expression of growth promoter gene *Igf2* was decreased in the alcohol-exposed E9.5 and E16.5 placentas. The expression of negative growth controller *H19* was significantly increased in the alcohol-exposed E9.5 embryos compared to controls, and conversely, a trend of decreased expression in alcohol-exposed E9.5 and E16.5 placentas were observed. Furthermore, increased *Snrpn* expression in alcohol-exposed E9.5 embryos was also detected. Our study indicates that albeit no alterations in the DNA methylation levels of studied sequences were detected by EpiTYPER, early PAE can affect the expression of imprinted genes in both developing embryo and placenta.

## Introduction

Prenatal alcohol exposure (PAE) can affect the development of embryo and cause a wide variety of birth defects and neuronal disorders. An umbrella term for the continuum of the variable effects of maternal alcohol consumption during pregnancy is fetal alcohol spectrum disorders (FASD). The most severe form of FASD is fetal alcohol syndrome (FAS), which is diagnosed by growth retardation, specific craniofacial abnormalities and functional or structural changes in central nervous system [1]. PAE is a leading cause of nongenetic mental retardation and birth defects in the Western world and the prevalence of FASD is estimated to range from 3 to 5% in Europe and North America to over 10% in South Africa [2].

The molecular mechanisms of PAE have been under extensive research, and the epigenetic variation induced in utero is a strong candidate mediator [3–5]. Previously, we have developed a mouse model of early PAE, based on maternal *ad libitum* ingestion of 10% (v/v) alcohol and took advantage of a mouse strain C57BL/6, which has a strong drinking preference for 10% alcohol [6,7]. The period of this moderate and chronic alcohol exposure is the first eight days of pregnancy (GD 0.5–8.5), from preimplantation to the beginning of neurulation. This is developmentally equivalent to the first three-four weeks of human pregnancy. By using this mouse model, we demonstrated for the first time that PAE could affect adult phenotype by altering the epigenotype of the early mouse embryo [3]. We discovered that alcohol exposure increases DNA methylation and probability of transcriptional silencing of an epigenetically sensitive allele *Agouti viable yellow* (*A*^*vy*^) [3] as well as changes the expression of several genes in liver [8] and hippocampi [9] of the offspring. The phenotype of the offspring was reminiscent of human FAS with craniofacial dysmorphology, postnatal growth restriction [3,8], and both structural [9] and functional [10] changes in the nervous system. Despite of postnatal growth restriction observed in this mouse model, prenatal growth restriction has not been detected in 16.5 embryonic days old (E16.5) embryos in our previous study [8]. However, owing to the relatively moderate alcohol exposure and subtle postnatal growth restriction in this model as well as prenatal growth restriction detected in previous mouse models with early and acute high-level alcohol-exposure [11,12], we cannot exclude the effect of early PAE on the regulation of growth.

**Fig 1:**
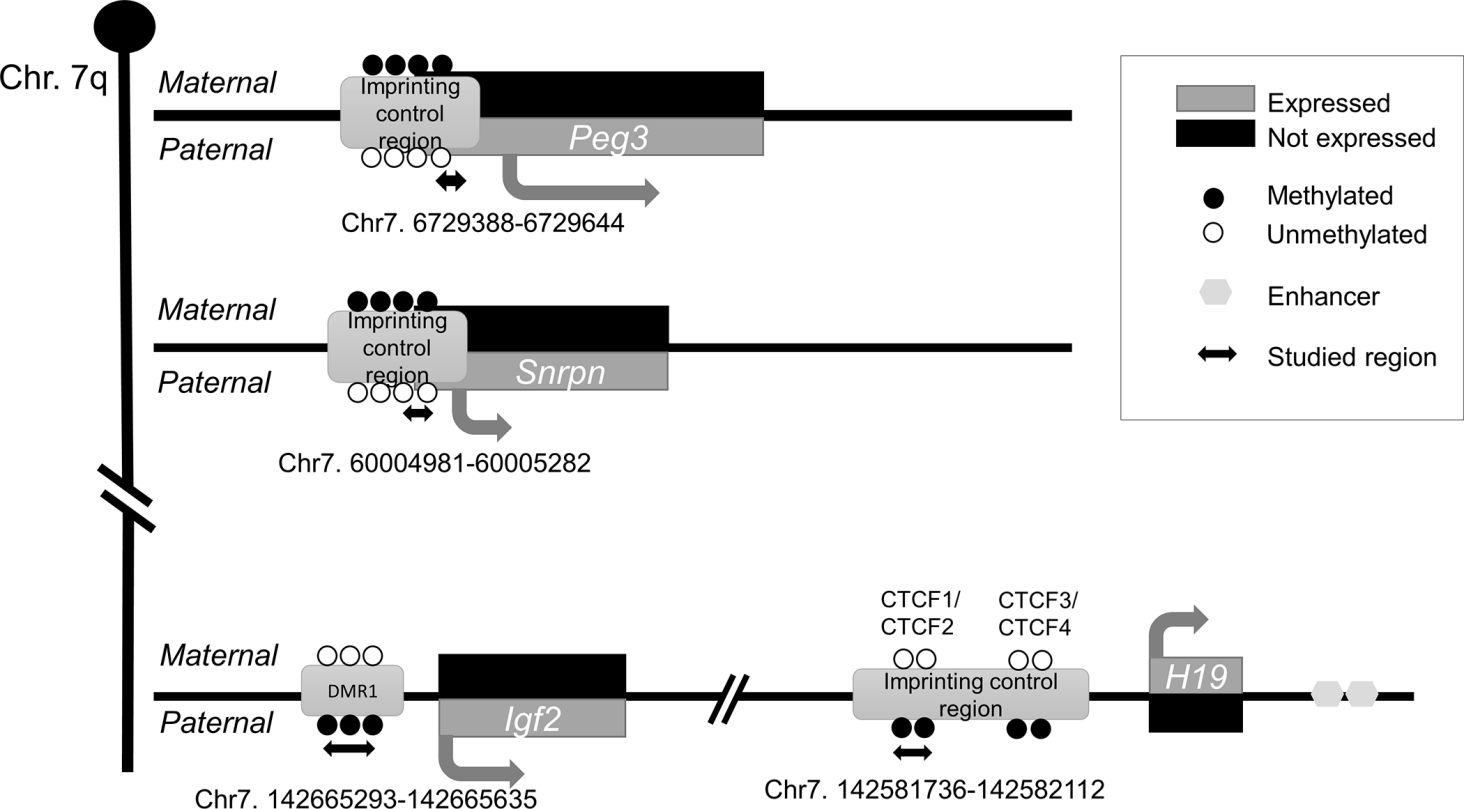
Schematic structure of studied imprinted genes on mouse chromosome 7q. Proximally, near the centromere, are *Paternally expressed gene 3 (Peg3)* and *Small Nuclear Ribonucleoprotein Polypeptide N (Snrpn)*, and distally *Insulin-like growth factor 2*/*H19 locus (Igf2/H19)*. Gray boxes illustrate maternally or paternally expressed active alleles and black boxes inactive alleles. Imprinting control regions (ICR) or differentially methylated regions (DMR) are marked with methylated (black) or unmethylated (white) balls. Arrows and sequences below the ICRs or DMR represent the regions of interest. Not drawn into scale.

Due to growth-restricted phenotype associated with PAE in both mouse and human, we focused on imprinted genes in this study. Imprinted genes are expressed actively in placenta and embryo, and they are important regulators of normal development [13]. They are epigenetically regulated by specific regulatory elements known as imprinting control regions (ICRs) or differentially methylated regions (DMRs), and organized in clusters containing both maternally and paternally expressed genes. One of the best-characterized imprinted domain is the *Insulin-like growth factor 2 (Igf2)*/*H19* locus on the distal mouse chromosome 7q (Fig 1). ICR in this locus controls the mono-allelic expression of the growth promoter gene *Igf2* [14] and negative growth controller *H19* [15]. Mouse ICR contains four binding sites for the methylation-sensitive zing-finger proteins, CCCTC-binding factors (CTCFs), which act as insulator when they bind on the hypomethylated maternal allele and promotes the *H19* expression. By contrast, *Igf2* is expressed on the methylated paternal allele in which the *H19* is repressed [16]. Furthermore, paternally expressed *Small Nuclear Ribonucleoprotein Polypeptide N* (*Snrpn*) [17] and *Paternally expressed gene 3* (*Peg3)* [18], both on proximal mouse chromosome 7, have been associated with growth (Fig 1).

In addition to environmental factors like maternal dietary restriction [19] or dietary compounds such as folate in human [20] and bisphenol A exposure in mouse [21], also PAE has been reported to affect the DNA methylation level of imprinted genes. An acute alcohol exposure during preimplantation period in mice decreased the weight of both E10.5 embryos and placentas, but the altered DNA methylation at the *H19* ICR was observed only in placentas [12]. Also, in both mouse [4,22] and human [23] genome-wide methylation studies PAE has been associated with methylation changes at the *H19*. Furthermore, in our previous study we observed genotype-specific decreased DNA methylation level at the *H19* ICR in the *IGF2/H19* locus in placentas of PAE newborns. A single nucleotide polymorphism rs10732516 in the sixth binding site for CTCF protein associated with the alcohol-induced alterations in DNA methylation profiles and head circumference in a parent-of-origin manner [5].

In this study, we used our mouse model to explore the effects of early PAE on the DNA methylation level and gene expression of four imprinted genes: *Insulin-like growth factor 2* (*Igf2*), *H19*, *Small Nuclear Ribonucleoprotein Polypeptide N* (*Snrpn*) and *Paternally expressed gene 3* (*Peg3*). We hypothesized that early, moderate PAE can change the developmental programming of growth in the beginning of embryonic development and that the programming can be detected as a growth-restricted phenotype in adolescent offspring. We examined potential alcohol-induced changes in DNA methylation at the *Igf2/H19* ICR (binding sites CTCF1 and CTCF2), *Igf2* DMR1, *Snrpn* ICR and *Peg3* ICR in E9.5 embryos and placentas. Alcohol-induced alterations in expression of these four imprinted genes were analyzed in E9.5 embryos and placentas. Furthermore, E16.5 placentas were analyzed to assess the stability of potential alcohol-induced expression changes. To determine alcohol-induced alterations in the global methylation level in placenta, we examined also DNA methylation at the long interspersed nuclear elements (Line-1) in the alcohol-exposed and control E9.5 placentas.

## Material and methods

### Mouse model

All the animals were handled and maintained with good animal practice according to the instructions, orders and ethical principles of EU-directive (European Union: 2010/63/EU, 2007/526/EY). All animal work was approved by the Animal Experiment Board in Finland (ESAVI/3312/04.10.03/2011, ESAVI/976/04.10.07/2013).

The mice in this study were inbred, genetically identical, C57BL/6J Rcc strain (Harlan, Netherlands). The experiments were performed in an animal house, where all environmental factors were standardized. The females (8–10 weeks old) were caged with males and the day of plugging was designated gestational day (GD) 0.5. The male was removed from the cage and the water bottle was replaced with a bottle containing 10% (v/v) alcohol. Strain C57BL/6J has a strong drinking preference for 10% alcohol [7] and voluntary maternal consumption strategy was used to decrease maternal stress. Alcohol and food were freely available for the first eight days of gestation. Alcohol was changed and consumption was measured every 24 hours. The average daily consumption of 10% alcohol during GD 0.5–8.5 was 3.5±0.5 (mean±SD) ml/mouse/day (or 17.1g±1.8g alcohol/kg body weight/day). In female mice, the consumption of 10% (w/v) alcohol at 14 g ethanol/kg body weight/day produces an average peak blood alcohol level of ~120mg/dl [24]. Blood alcohol level of 0.12% is a realistic human exposure of alcohol, which makes this a plausible model to study the effects of chronic and moderate alcohol exposure. Alcohol bottle was replaced with a tap water bottle on the final day of exposure (GD 8.5). Tap water and food were freely available for control females through the whole procedure.

At embryonic day 9.5 (E9.5) or 16.5 (E16.5) dams were sacrificed by carbon dioxide followed by cervical dislocation. At E9.5 both embryos and placentas, and at E16.5 only placentas were dissected carefully removing maternal decidua. All the samples were snap frozen in liquid nitrogen and stored at -80°C.

### DNA methylation analysis

Methylation analysis was done for five control and five alcohol-exposed E9.5 embryos and placentas. Both the embryo and placenta were studied from the same individual, except one control and one alcohol-exposed sample were from different origin. The samples in both control and alcohol-exposed groups were from two or three litters.

Genomic DNA was extracted by traditional phenol-chloroform protocol or by Allprep DNA/RNA/miRNA kit (Qiagen, Valencia, CA, USA). Sodium bisulphite conversion of extracted DNA (1000 ng) was carried out using the EZ methylation kit (Zymo Research, Irvine, CA, USA). Three PCR reactions were performed for each sample by using the HotStar PCR kit (Qiagen, Valencia, CA, USA) or PCR enzyme optimized for EpiTYPER (Seqenom) in a 10 μl reactions according to the provider’s instructions.

MassARRAY EpiTYPER (SEQUENOM Inc.) technique, based on matrix assisted laser desorption ionization time-of-flight (MALDI-TOF) mass spectrometry, was used to detect alcohol-induced changes in the methylation level of the imprinted genes. Amplicons were designed for the sequences in the imprinting control region or the differentially methylated region of the studied imprinted genes *Igf2*, *H19*, *Snrpn and Peg3*: *Igf2/H19* ICR (CTCF1/CTCF2) (chromosome 7: 142581736–142582112; GRCm38.p3), *Igf2* DMR1 (chromosome 7: 142665293–142665635; GRCm38.p3), *Snrpn* ICR (chromosome 7: 60004981–60005282; GRCm38.p3) and *Peg3* ICR (chromosome 7: 6729388–6729644; GRCm38.p3). All the primer sequences for imprinted genes were designed with EpiDesigner software (SEQUENOM: http://www.epidesigner.com/; T-reaction). Primers for Line-1 was chosen from previous study [25]. The EpiTYPER analysis was done in triplicates for amplicons: *Igf2* DMR1, *Peg3* and Line-1, and the three PCR products were pooled for amplicons *Igf2/H19* ICR and *Snrpn*. A strict quality control was performed prior to analysis. All CpG-units that expressed too low, high mass or silent peak overlap of the units, were discarded. CpG-units, unable to analyze separately due to their close location, were analyzed together as a mean methylation value. Furthermore, technical replicates showing > 5% difference from the median value were discarded and only two successful replicates were analyzed.

### Expression study by quantitative PCR

Quantitative PCR was used to study the alcohol-induced effects on expression of imprinted genes *Igf2*, *H19*, *Snrpn* and *Peg3* in embryos (E9.5) and placentas (E9.5 and E16.5). Expression analysis was done for 10 control and 10 alcohol-exposed samples. Four different litters (in both control and alcohol-exposed groups) were used in E9.5 samples, whereas two litters (controls) or four litters (alcohol-exposed) were used in E16.5 samples.

Total RNA was extracted by Allprep DNA/RNA Mini kit (kit_1) (8 E9.5 embryos and placentas, E16.5 placentas: 4 ethanol-exposed and 4 controls) or Allprep DNA/RNA/miRNA kit (kit_2) (12 E9.5 embryos and placentas, E16.5 placentas: 6 ethanol-exposed and 6 controls) (Qiagen, Valencia, CA, USA). RNA was DNAse treated (RQ1 RNase-Free DNase, Promega, Madison, WI, USA) and transcribed to cDNA by using the iScript cDNA Synthesis Kit (Bio-Rad Laboratories, Hercules, CA, USA). The qPCR reaction conditions were as specified by SYBR^®^ Green PCR Master Mix according to the manufacturer’s protocol (Applied Biosystems, Foster City, CA, USA). Primers for *Igf2* [26], *H19* [27], *Snrpn* [28] and *Peg3* [29] were chosen from previous studies. Housekeeping gene *Ribosomal protein*, *large*, *P0* (*Rplp0*) was used as a reference gene (S1 Fig) [30]. Samples were analyzed in triplicates and the individual samples (both control and alcohol-exposed) were normalized by adding one same control sample on each plate. If the Ct-value was <0.5 between the triplicates, all of them were accepted for further analysis. The relative expression of studied genes was obtained by normalizing the Ct-values of the gene of interest to the Ct-values of housekeeping gene *Rplp0*. According to the Livak and Schmittgen method, the control samples were used as a calibrator to alcohol-exposed samples to calculate the ΔΔCt and fold changes were calculated as 2^(-ΔΔCt) [31]. To assure the effectiveness and reliability of each qPCR plate, a standard curve was added.

To rule out the possible contamination of the maternal decidua in placentas prior to expression studies, the expressions of *Tissue factor pathway inhibitor 2* (*Tfpi2*) and *Adenosine monophosphate deaminase 3* (*Ampd3)* genes were examined by qPCR as described in Okae et al. 2012 [32]. These genes show a high expression in the placental cells of maternal side. This examination was done for four E16.5 placentas. Two placentas without maternal decidua were compared to two placentas with maternal decidua (S2 Fig).

### Sex determination

The sex of the embryos was determined from DNA with *Sex-determining region Y* (*Sry*) and *Interleukin 3* (*Il3*) genes by PCR [33]. Primers of *Sry* bind to Y-chromosome specific *Sry* gene, naturally observed in males. *Il3* gene is used as a control to the reaction, since it is found from both sexes. PCR was done by using AmpliTaq Gold^®^ DNA polymerase kit, according to the manufacturer’s protocol (Thermo Fisher, Waltham, MA, USA). Both primer pairs (*Sry*, *Il3*) were included to a single reaction. This way two PCR products were visible for male embryos (*Sry*, *Il3*) and one for females (*Il3*).

Details regarding the primers and protocols of DNA methylation, gene expression and sex determination studies are found in supplementary material (S1 Table).

### Statistical analysis

Statistical analyses were conducted using IBM SPSS Statistics 24. All data is expressed as the means ± standard deviation for a normal distribution. Normality of the data was tested with Shapiro-Wilk’s test. Potential outliers were tested with Grubb’s test. To compare methylation level of single CpG sites in studied regions between alcohol-exposed and control samples, multivariate Hotelling’s T^2 test was used. In expression analysis, to eliminate the possible interaction effect caused by the two different RNA extraction kits, two-way ANOVA was used. If interaction was detected, differences between two groups were compared by two-way Student’s t-test.

## Results

### Methylation analysis

**Fig 2:**
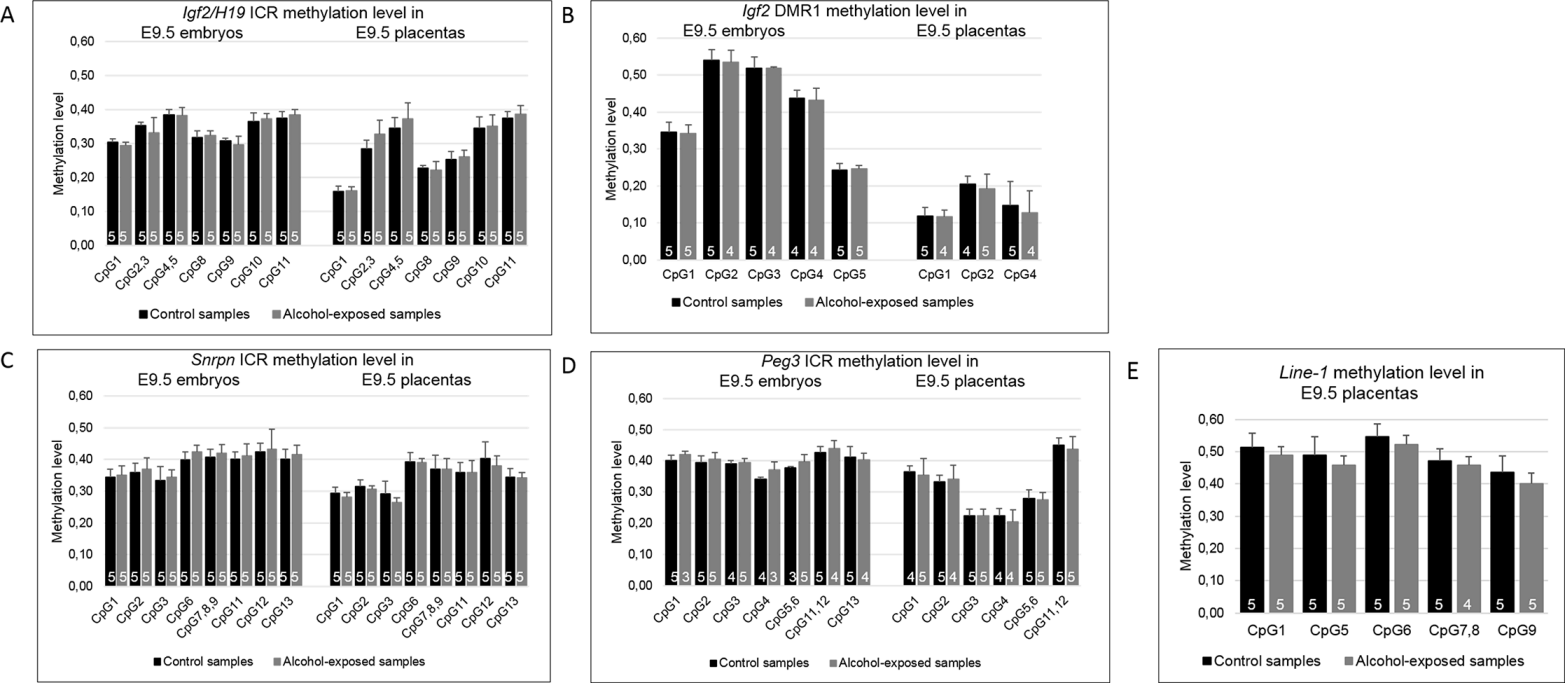
**DNA methylation levels of CpG sites at the *Igf2*/*H19* ICR (A), *Igf2* DMR1 (B), *Snrpn* ICR (C), *Peg3* ICR (D), and Line-1 (E) in 9.5 embryonic days old (E9.5) control and alcohol-exposed embryos and placentas.** Control samples are colored in black and alcohol-exposed samples in gray. The numbers of samples are presented in columns. CpG sites 3 and 5 at *Igf2* DMR as well as CpG site 13 at *Peg3* ICR in E9.5 placentas have been excluded since no methylation value could be detected by EpiTYPER. Error bars denote the SD.

To explore alcohol-induced changes in the differentially methylated regions (DMR) or imprinting control regions (ICR) of the candidate genes we used the EpiTYPER method. However, no significant DNA methylation changes were observed between alcohol-exposed and control E9.5 embryos or placentas in any of the studied regions: *Igf2/H19* ICR, *Igf2* DMR1, *Snrpn* ICR, *Peg3* ICR (Fig 2, S2 and S3 Tables).

We also examined DNA methylation at the long interspersed nuclear elements (Line-1) in the alcohol-exposed and control E9.5 placentas to determine alcohol-induced alterations in the global methylation level. However, no significant methylation changes were observed (Fig 2E).

### Expression analysis

**Fig 3:**
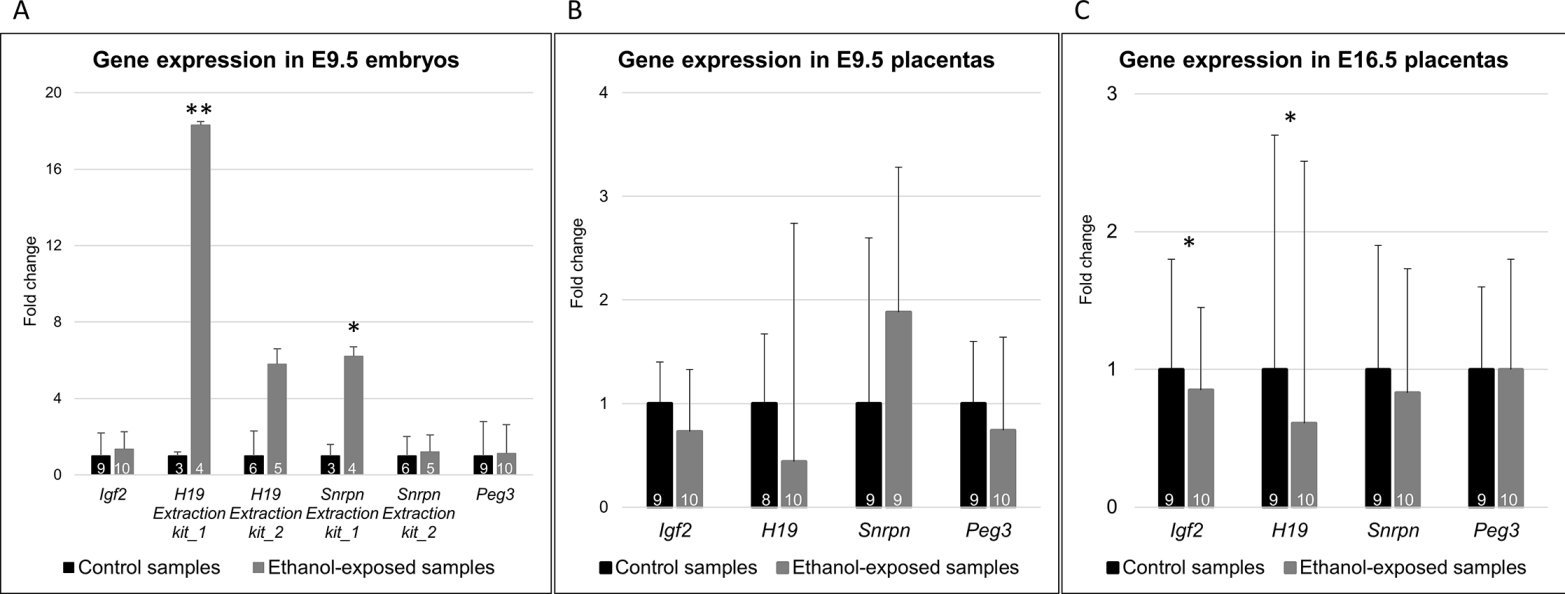
Expression levels of imprinted genes in control and alcohol-exposed 9.5 embryonic day old (E9.5) embryos and placentas as well as E16.5 placentas. *Igf2*, *H19*, *Snrpn* and *Peg3* expressions in control and alcohol-exposed E9.5 embryos (A), E9.5 placentas (B) and in E16.5 placentas (C). Control samples are colored in black and alcohol-exposed samples in gray. The numbers of samples are presented in columns (expression of *Snrpn* in one embryo and one E9.5 placenta samples was not detected). Error bars denote the SD. P-value; (A) two-way Student’s t-test: *p<0.05, **p<0.001, (C) Two-way ANOVA: *p<0.05.

We used quantitative PCR method to observe potential expression differences in imprinted genes *H19*, *Igf2*, *Snrpn* and *Peg3* between ethanol-exposed and control embryos and placentas (Fig 3, S4 Table). Since two different RNA extraction methods were used in the study (see materials and methods), we eliminated the possible interaction effect of the extraction kit by using a two-way ANOVA. Two outliers (E9.5 ethanol-exposed embryo and E9.5 control placenta) were excluded from the data.

#### E9.5 embryos

We observed up-regulated gene expression of *H19* and *Snrpn* in E9.5 embryos. However, since there was an interaction effect with the extraction kit (F_1,14_ = 11.9, p = 0.004; F_1,14_ = 8.5, p = 0.01, respectively), we divided the samples into two groups according to the kit. A significant difference between alcohol-exposed and control samples was detected only in the group of extraction kit_1 (*H19*: p<0.001; *Snrpn*: p = 0.002, two-tailed Student’s t-test) (Fig 3A). No difference in *Igf2* (F_1,15_ = 0.14, p = 0.7) or *Peg3* (F_1,15_ = 0.08, p = 0.8) expression between the alcohol-exposed and control samples was detected. Only *Peg3* had a significant difference between the extraction methods (*Peg3*: F_1,15_ = 28.3, p<0.001; *Igf2*: F_1,15_ = 4.2, p = 0.06), although the interaction effect was not significant (*Peg3*: F_1,15_ = 1.3, p = 0.3) (Fig 3A).

#### E9.5 placentas

In addition to embryos, we explored alcohol-induced expression changes in the placental tissue. Even though we detected minor expression changes in all studied genes, the difference between alcohol-exposed and control samples was not significant (*Igf2*: F_1,15_ = 4.1, p = 0.06; *H19*: F_1,14_ = 0.9, p = 0.4; *Snrpn*: F_1,14_ = 1.8, p = 0.2; *Peg3*: F_1,15_ = 2.1, p = 0.2) (Fig 3B).

There were no differences between the extraction methods in *H19* (F_1,14_ = 1.1, p = 0.3), but significant differences were detected in *Igf2*, *Snrpn* and *Peg3* (F_1,15_ = 12.2, p = 0.003; F_1,14_ = 14.2, p = 0.002; F_1,15_ = 11.9, p = 0.004, respectively). However, the interaction effects were not significant (F_1,15_ = 0.09, p = 0.7; F_1,14_ = 0.7, p = 0.4; F_1,15_ = 0.6, p = 0.5, respectively).

#### E16.5 placentas

To see if the observed expression changes in the E9.5 placentas are stable throughout the pregnancy, we explored the gene expression of the same genes in E16.5 placentas as well. Similarly, as in E9.5 placentas, *H19* and *Igf2* expression remained decreased in the alcohol-exposed E16.5 placentas compared to controls (F_1,15_ = 9.8, p = 0.007; F_1,15_ = 5.2, p = 0.04, respectively) (Fig 3C). Interestingly, *Snrpn* expression was decreased in the alcohol-exposed E16.5 placentas (F_1,15_ = 1.6, p = 0.2), which is in contrast to the E9.5 embryos and E9.5 placentas, in which the expression was increased. Similarly, as in E9.5 embryos and E9.5 placentas, *Peg3* did not show expression change in the alcohol-exposed E16.5 placentas compared to controls (F_1,15_ = 0.3, p = 0.6).

Despite the differences in *Igf2*, *H19*, and *Snrpn* between the two extraction methods (F_1,15_ = 53.5, p<0.001; F_1,15_ = 135.2, p<0.001; F_1,15_ = 23.4, p<0.001, respectively), there were no interaction effects (F_1,15_ = 1.5, p = 0.2; F_1,15_ = 0.1, p = 0.7; F_1,15_ = 0.003, p = 0.96, respectively). There was no difference in *Peg3* between the extraction methods (F_1,15_ = 0.6, p = 0.5).

#### *H19/Igf2* expression ratio in each embryo and placenta

In previous studies, it has been shown that hypermethylation at *H19* ICR in Beckwith-Wiedemann syndrome with fetal over-growth leads to overexpression of *IGF2* and downregulation of *H19*, and conversely, hypomethylation of *H19* ICR in Silver-Russell syndrome with growth restriction leads to downregulation of *IGF2* and biallelic expression of *H19* [34,35]. In addition, in our previous human study, we observed significantly increased expression of *H19* in relation to *IGF2* when comparing all alcohol-exposed placentas to unexposed controls [5]. Therefore, we decided to explore if prenatal alcohol exposure has affected the DNA methylation level of the locus and consequently the regulation of *Igf2* and *H19*. We calculated the expression ratios of *H19* and *Igf2* in each mouse embryo and placenta and observed that *H19* expression was not increased in relation to *Igf2* in alcohol-exposed embryos or placentas in this study.

### Effects of sex

Since previous studies have shown that environmental factors can affect DNA methylation as well as gene expression in a sex-specific manner [36,37], we also explored sex-specific effects. Owing to the small sample size in DNA methylation analysis, we did not explore sex-specific effects on methylation level. We calculated possible sex-specific effects on gene expression in alcohol-exposed samples, however, no differences between males and females were observed (S5 Table).

## Discussion

Growth restriction is one of the major consequences of prenatal alcohol exposure (PAE). Owing to the prenatal growth restriction observed in previous mouse models with early, acute PAE [11,12], as well as alcohol-induced postnatal growth restriction in our mouse model of moderate and chronic PAE, we focused on growth-related imprinted genes in this study. *Igf2/H19* locus is one of the most well characterized imprinted region and crucial for normal growth of both embryo and placenta. We explored alcohol-induced alterations in DNA methylation levels of two CTCF binding sites, CTCF1 and CTCF2, in E9.5 embryos and placentas. However, we did not observe differences between control and alcohol-exposed samples. Although previous mouse studies have shown decreased methylation level at the *H19* ICR in placenta [12], as well as in the brain [4,38] and sperm [38] of *in utero* exposed offspring, the results have not been consistent in human studies. PAE associated decreased methylation at the *H19* has been observed in buccal epithelial cells of Canadian FASD children [23], but neither in blood samples of South African FAS children [36] nor in cord blood of newborns in six general population based cohorts [39]. In addition to different doses, timing and the length of exposure periods in experiments, the explanation for variability of findings could be tissue-specificity of changes: similar altered methylation has not been detected in alcohol-exposed placentas and embryos in the same study [12].

In our previous human study, a polymorphism rs10732516 G/A in the sixth binding site for CTCF protein associated with the alcohol-induced alterations in DNA methylation level in a parent-of-origin manner [5]. We observed decreased placental methylation level in the paternal allele only in paternal A and maternal G (patA/matG) genotype of the two studied heterozygous genotypes. In addition to methylation, also genotype-specific differences in placental gene expression and phenotype of exposed newborns were observed [5], which makes the comparisons between human and mouse studies difficult. Nevertheless, as in G/G and patA/matG genotypes in the alcohol-exposed human placentas, a similar trend of decreased *Igf2* expression was detected in alcohol-exposed mouse E9.5 and E16.5 placentas in this study, which have also been seen after acute alcohol exposure during the GD9 in both embryo and placenta [40]. We could speculate that because allele A deletes a CpG site for a methyl group at the human *H19* ICR, the G/G could be the original genotype and the most comparable to the mouse study.

The expression of a negative growth controller *H19* was increased in alcohol-exposed embryos when comparing to controls, which could explain the growth restricted phenotype of offspring. The precise biological role of *H19* is unknown, but it has been suggested that mir-675 encoded by *H19*, has a role in restricting growth or cell proliferation by downregulating *Insulin-like growth factor 1 receptor* expression [41,42]. However, the expression level of mir-675 was not measured in this study. By contrast, a trend of decreased *H19* expression was observed in alcohol-exposed E9.5 and E16.5 placentas, which is not consistent with the slightly increased *H19* expression of G/G genotype in our human placentas [5]. Furthermore, *H19/Igf2* ratio in alcohol-exposed placentas or embryos was not altered in this mouse study, whereas it was significantly increased in our human study. The reason could be a moderate alcohol-exposure in this mouse model compared to our human samples, the timing of exposure, too small sample size to detect subtle changes or differences in the effects of PAE on the regulation of *IGF/H19* locus between mouse and human.

The decreased expression of maternally imprinted *Snrpn* has been linked to the imprinting disorder, Prader-Willi syndrome [43]. Typical characteristics of this syndrome are mild intellectual disability, low birth weight as well as behavioral and learning problems [17], which are features observed also in FASD children. We did not detected changes in methylation level at the *Snrpn* ICR, which is consistent with an earlier study, where low dose of alcohol administration did not affect the *Snrpn* ICR methylation profile in any tissue in mouse [38]. However, we saw a significantly increased *Snrpn* expression in alcohol-exposed E9.5 embryos compared to controls. Interestingly, similar result has been shown before, when consequences of paternal alcohol exposure has been studied: exposure of male mice to high dose of ethanol decreases methylation level at *Snrpn* ICR and increases the *Snrpn* expression in the cerebral cortex of F1 offspring [44].

*Paternally expressed gene 3* is highly expressed in hypothalamus, which is involved in control of maternal-caring behaviors and milk provision. Altered expression has been associated with defects in milk-suckling behaviors and consequently weaker mouse offspring due to insufficient uptake of milk [45,46] as well as growth restriction [47]. However, in our mouse study we did not detect altered *Peg3* expression in alcohol-exposed embryos or placentas. In the South African FAS study the methylation level of *Peg3* ICR was significantly decreased in the blood samples of FAS children compared to controls [36]. However, we did not observe any changes in methylation levels at the *Peg3* ICR, which is consistent with the study by Stouder and colleagues (2011) [38].

As a conclusion, we did not observe any PAE associated changes in DNA methylation levels of imprinted genes in this mouse model. Since alcohol can cause allele-specific methylation changes in imprinted regions [5,12], MassARRAY EpiTYPER, which measures total methylation level, could be too robust method to detect such alterations. By using traditional bisulfite sequencing and two different mouse strains it could be possible to see small allele-specific methylation changes in the candidate sequences. The moderate alcohol exposure in this model causes highly variable phenotype for the offspring and thus also a larger sample size is probably needed to reveal subtle changes in methylation level. Or perhaps this early exposure is incapable to change this specific level of epigenetic regulation in these particular regions we analyzed. Although earlier studies have shown that several environmental exposures like PAE [4,5,12,23], in vitro fertilization [48], [49] and nutrition [50] can affect the methylation level of ICRs, these specific regions could be more resistant to environmental factors compared to DMRs. The methylation of ICRs is established already in germline and protected from dynamic epigenetic reprogramming after fertilization by several factors [51]. Thus, in the future studies we should focus on DMRs, in which methylation profile will be formed in the beginning of embryonic development, concurrently with the alcohol exposure in this mouse model.

However, we observed changes in gene expressions, which associated with PAE. The most significant findings, decreased expression of the growth promoter *Igf2* in alcohol-exposed placentas as well as increased expression of negative growth controller *H19* in alcohol-exposed embryos, are consistent with the growth-restricted phenotype of the offspring in this mouse model. This emphasizes the significance of *Igf2/H19* locus and its regulation in the PAE phenotype, and encourage us to study more profoundly the function of this complex locus in the future.

## Supporting information

S1 Fig Gene expression of housekeeping gene. Ethanol-exposed samples normalized to control samples to obtain fold difference of *Rplp0*. Columns present average value and standard deviation of the ethanol-exposed samples. Number of the samples is presented in the column.
(TIF)

S2 Fig Detection of maternal decidua contamination. Gene expression of *Ampd3* and *Tfpi2* in four E16.5 placentas. Two placentas with maternal decidua (gray) were used as controls to two placentas without decidua (black). Figure presents the fold difference of the gene expression.
(TIF)

S1 Table Details regarding the primers and protocols. (A) Primers for EpiTYPER and expression studies. (B) Protocol for EpiTYPER PCR reaction.
(TIF)

S2 Table EpiTYPER result. DNA methylation levels of *Igf2/H19* ICR, *Igf2* DMR1, *Snrpn* ICR, *Peg3* ICR and *Line-1* in control and alcohol-exposed placentas by EpiTYPER method. Methylation average values and standard deviations (±) of CpG units are presented in the table.
(TIF)

S3 Table Comparison between single CpG sites in the studied ICR/DMR region with Hotelling’s T^2 test. (TIF)

S4 Table Gene expression result. Relative gene expression (dCT values) of *Igf2*, *H19*, *Snrpn* and *Peg3* in E9.5 embryos and placentas as well as E16.5 placentas by qPCR. Average values and standard deviations (±) of control and ethanol-exposed samples are presented in the table.
(TIF)

S5 Table Sex-specific differences of gene expression in ethanol-exposed samples. There was no significant difference between male and female ethanol-exposed samples. P-value: Mann-Whitney.
(TIF)
