# Supplementary figures and images for: Early prenatal alcohol exposure alters imprinted gene expression in placenta and embryo in a mouse model
Source: PLoS One. 2018 May 15;13(5):e0197461. doi: 10.1371/journal.pone.0197461 (PMC5953443; doi:10.1371/journal.pone.0197461)

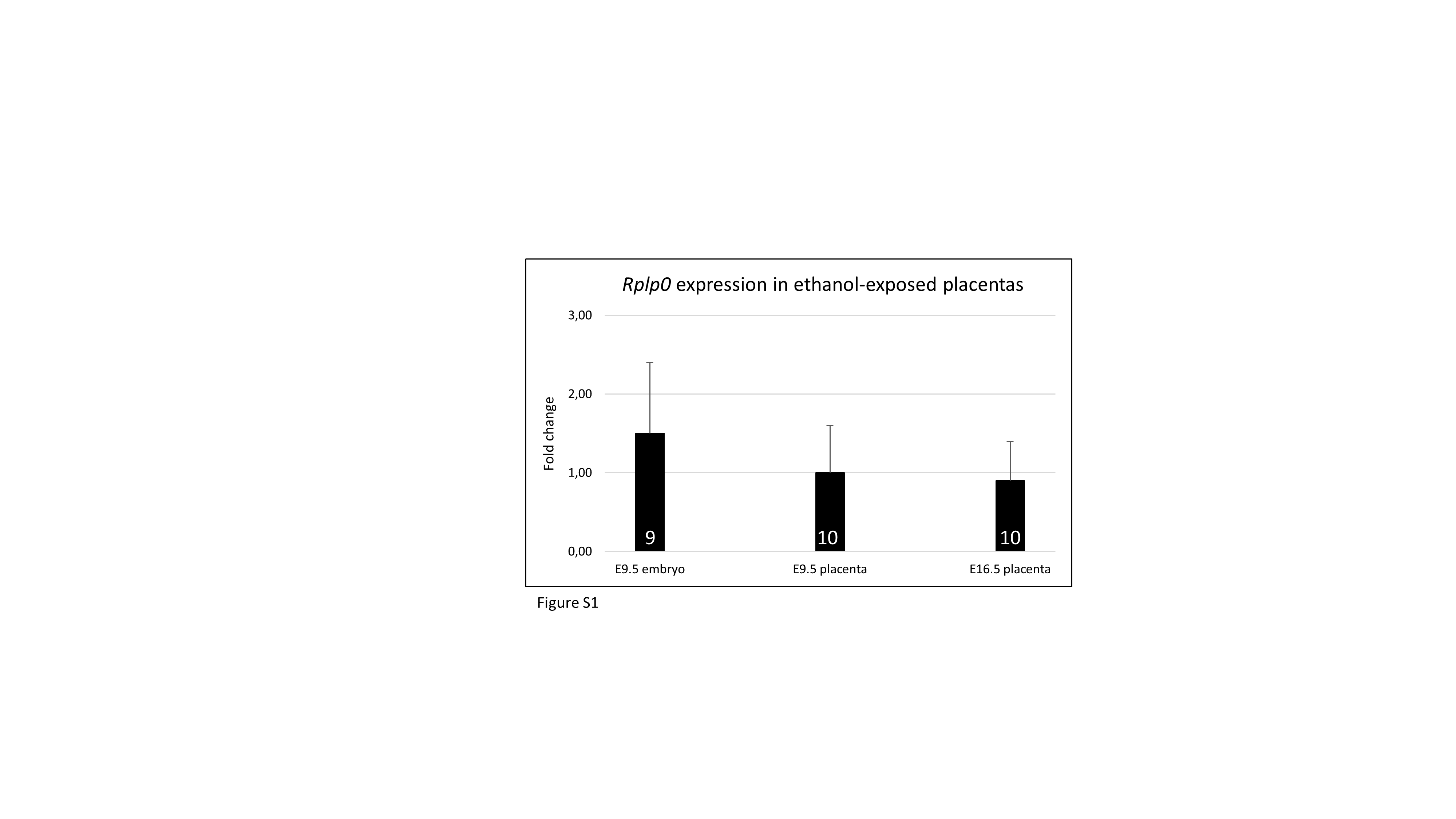

Supplement: S1 Fig — Ethanol-exposed samples normalized to control samples to obtain fold difference of Rplp0. Columns present average value and standard deviation of the ethanol-exposed samples. Number of the samples is presented in the column. (TIF) [file pone.0197461.s001.tif]

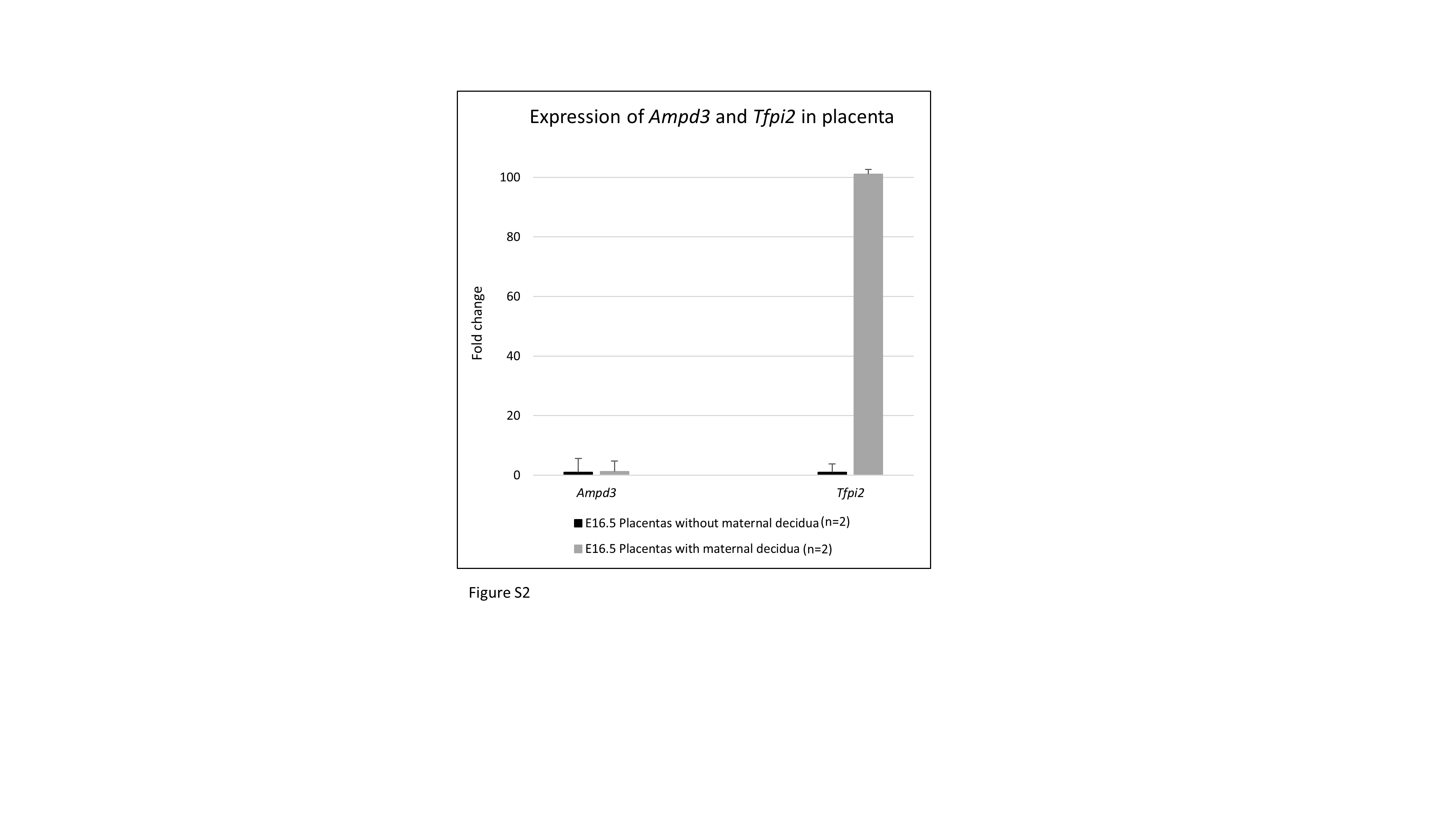

Supplement: S2 Fig — Gene expression of Ampd3 and Tfpi2 in four E16.5 placentas. Two placentas with maternal decidua (gray) were used as controls to two placentas without decidua (black). Figure presents the fold difference of the gene expression. (TIF) [file pone.0197461.s002.tif]

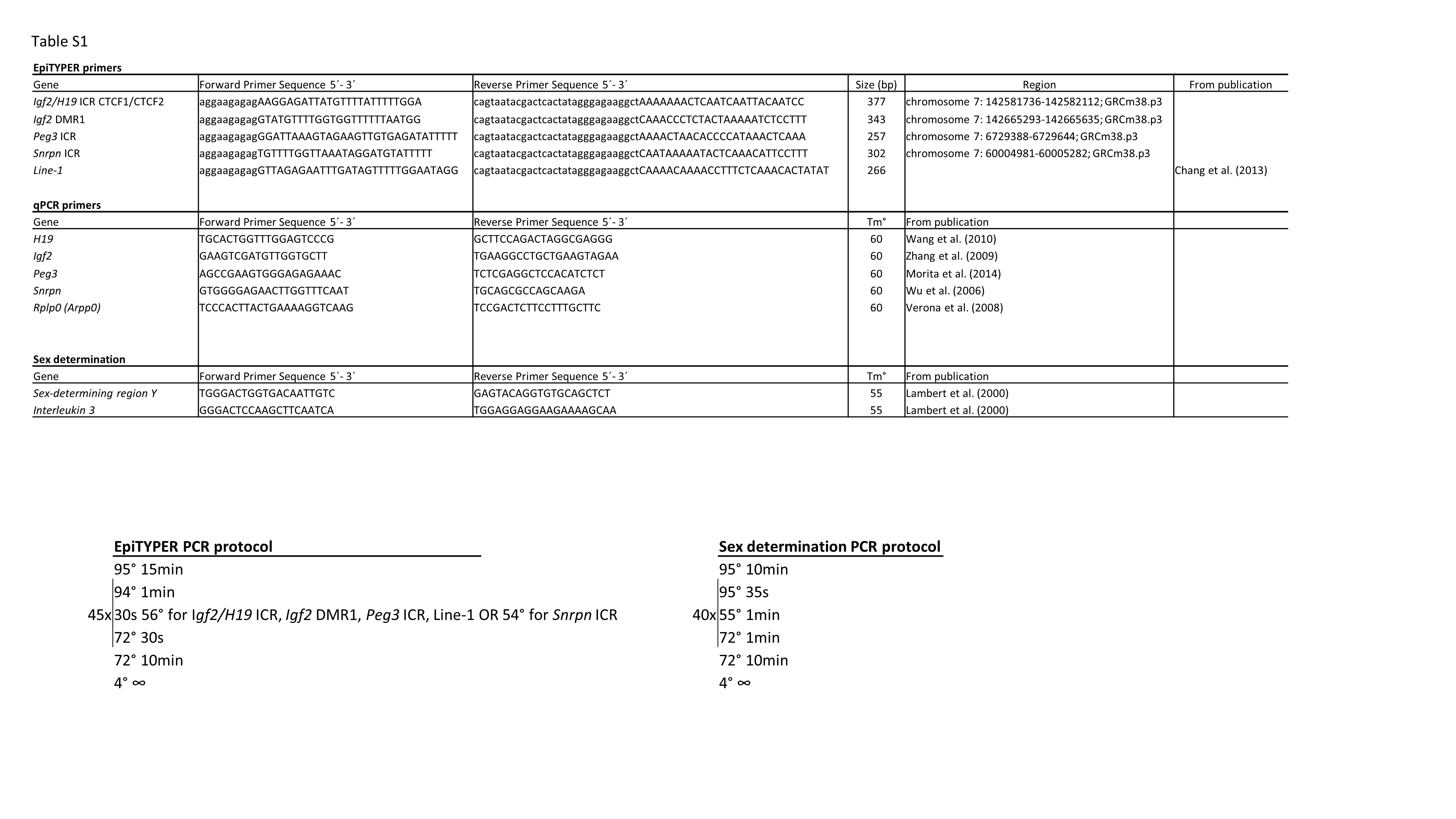

Supplement: S1 Table — (A) Primers for EpiTYPER and expression studies. (B) Protocol for EpiTYPER PCR reaction. (TIF) [file pone.0197461.s003.tif]

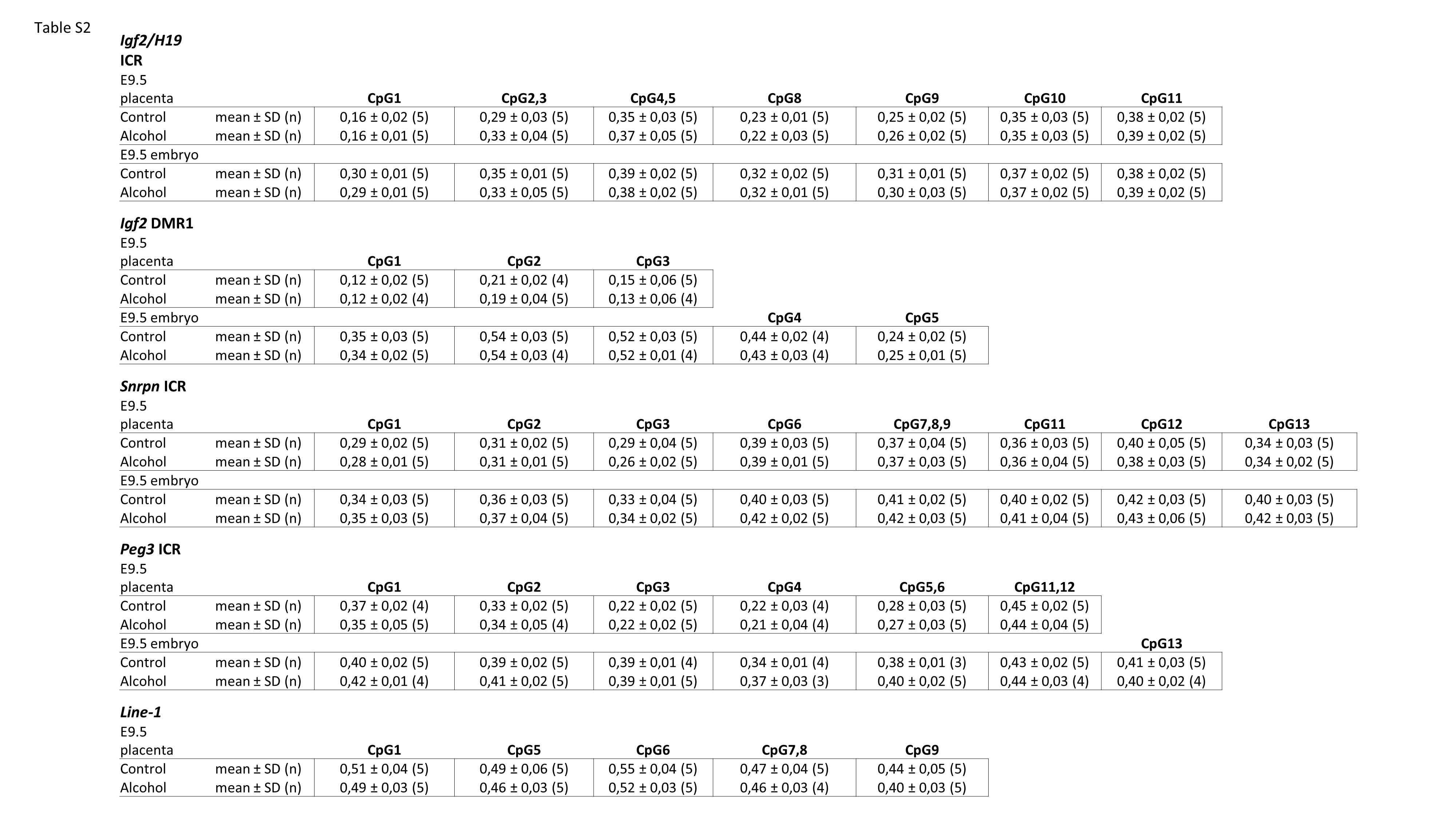

Supplement: S2 Table — DNA methylation levels of Igf2/H19 ICR, Igf2 DMR1, Snrpn ICR, Peg3 ICR and Line-1 in control and alcohol-exposed placentas by EpiTYPER method. Methylation average values and standard deviations (±) of CpG units are presented in the table. (TIF) [file pone.0197461.s004.tif]

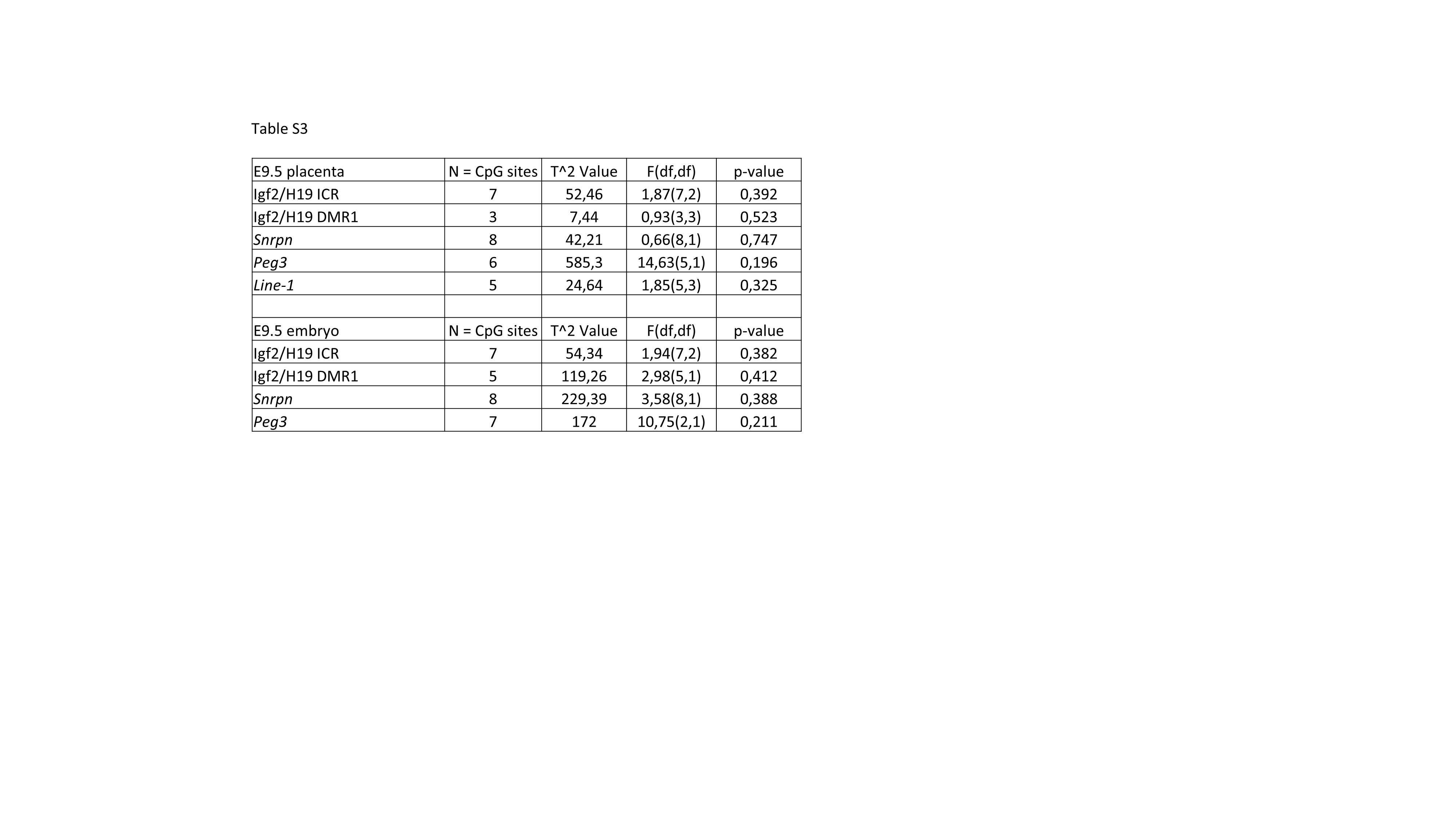

Supplement: S3 Table — (TIF) [file pone.0197461.s005.tif]

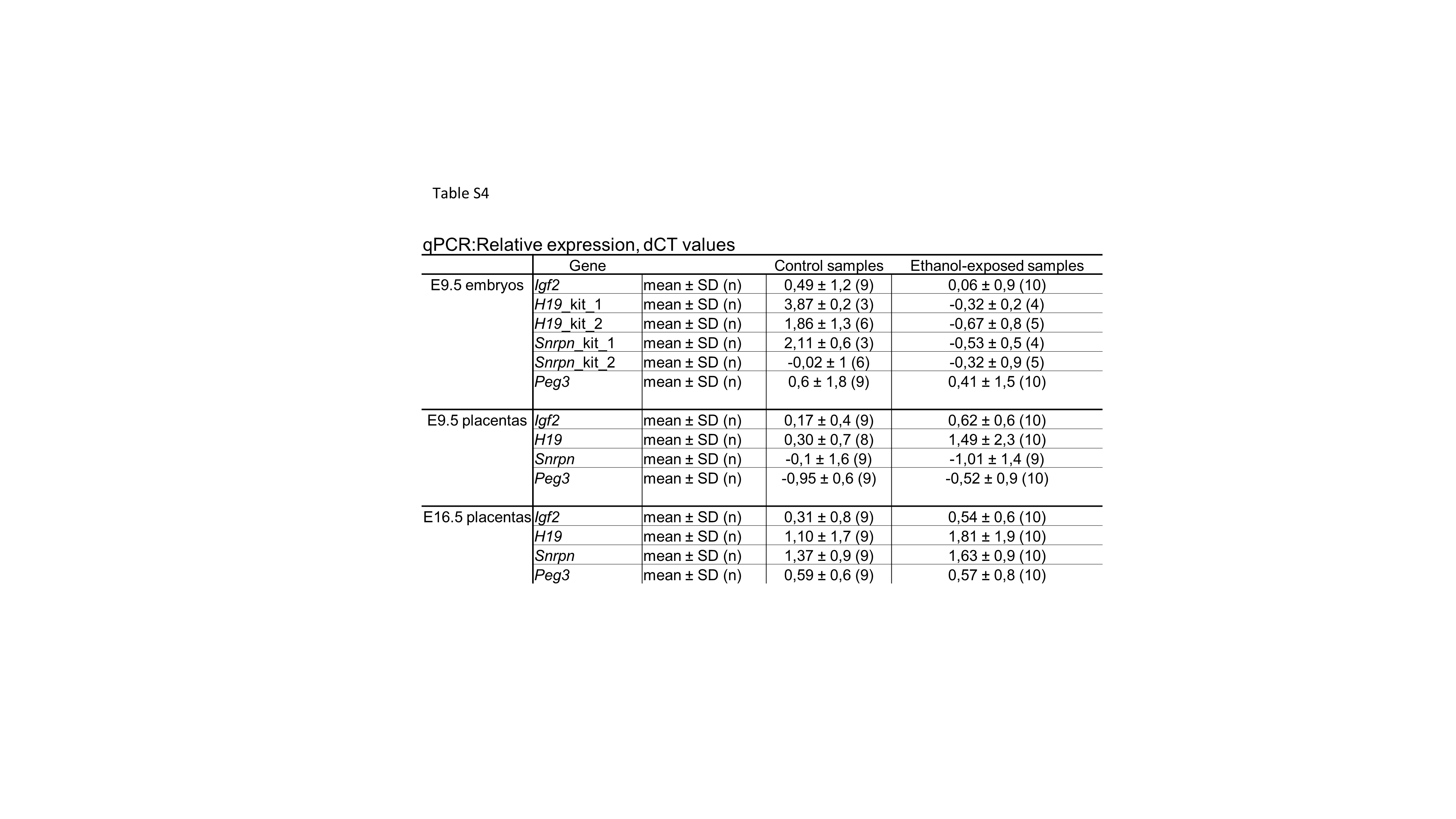

Supplement: S4 Table — Relative gene expression (dCT values) of Igf2, H19, Snrpn and Peg3 in E9.5 embryos and placentas as well as E16.5 placentas by qPCR. Average values and standard deviations (±) of control and ethanol-exposed samples are presented in the table. (TIF) [file pone.0197461.s006.tif]

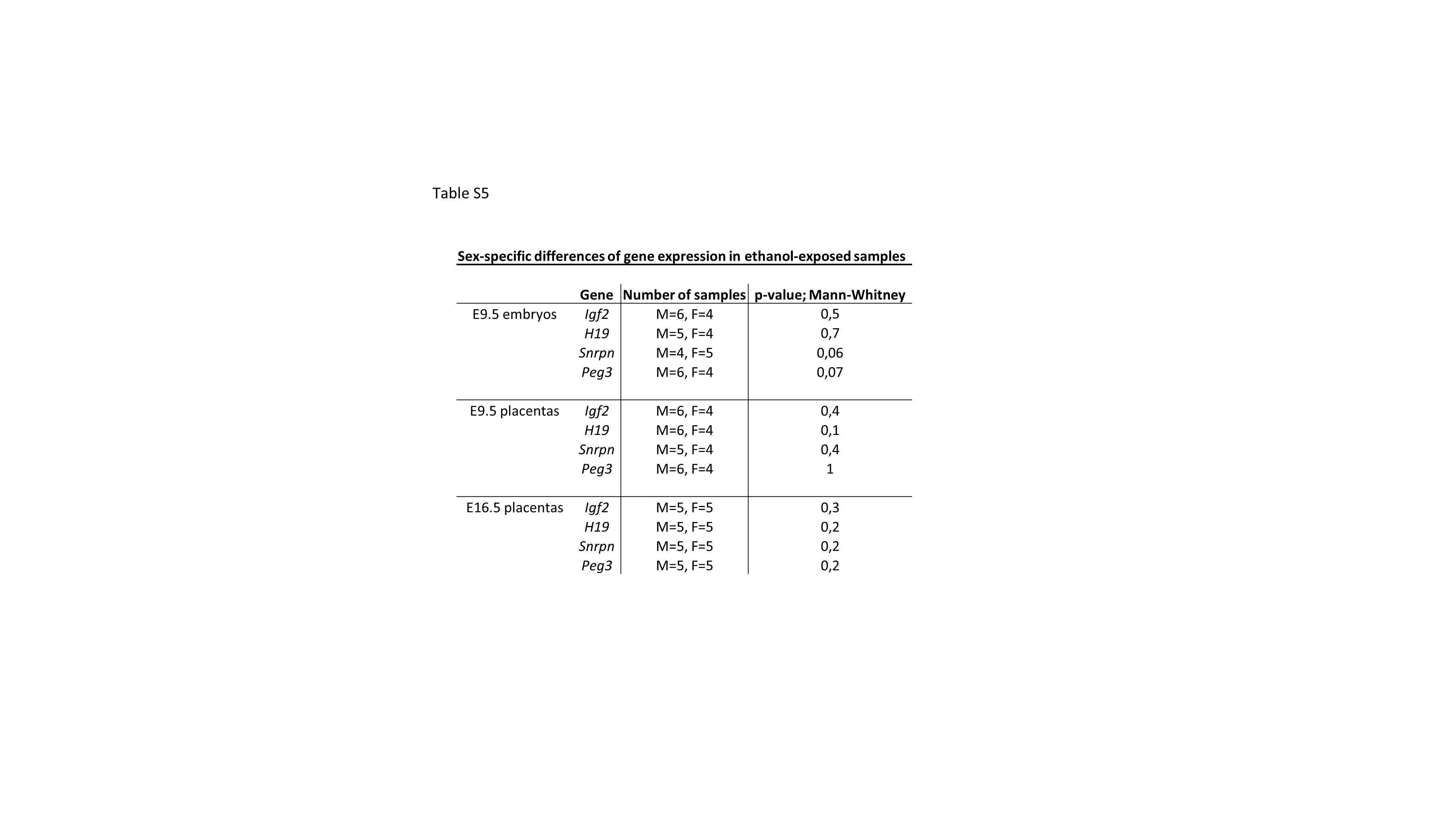

Supplement: S5 Table — There was no significant difference between male and female ethanol-exposed samples. P-value: Mann-Whitney. (TIF) [file pone.0197461.s007.tif]
